# Enhanced Sensitivity to Low Dose Irradiation of ApoE−/− Mice Mediated by Early Pro-Inflammatory Profile and Delayed Activation of the TGFβ1 Cascade Involved in Fibrogenesis

**DOI:** 10.1371/journal.pone.0057052

**Published:** 2013-02-22

**Authors:** Virginie Monceau, Lydia Meziani, Carine Strup-Perrot, Eric Morel, Magret Schmidt, Julia Haagen, Brigitte Escoubet, Wolfgang Dörr, Marie-Catherine Vozenin

**Affiliations:** 1 INSERM U1030, Villejuif, France; 2 Université Paris Sud, Kremlin-Bicêtre, France; 3 Institut Gustave Roussy, Villejuif, France; 4 Institut de la Radioprtection et de Sureté Nucléaire, LRTE/SRBE/DRPH, Fontenay-aux Roses, France; 5 UMR-S 769, INSERM, Chatenay Malabry, France; 6 Department of Radiotherapy and Radiation Oncology, Medical Faculty Carl Gustav Carus, Technological University Dresden, Dresden, Germany; 7 Département de Physiologie, Explorations Fonctionnelles, Assistance Publique-Hôpitaux de Paris, Hôpital Bichat, Paris, France; 8 Université Paris 7 Diderot, Paris, France; 9 INSERM U872, Paris, France; 10 Department of Radiation Oncology and Christian Doppler Laboratory for Medical Radiation Research in Radio oncology Medical University, Vienna, Austria; Temple University, United States of America

## Abstract

**Aim:**

Investigating long-term cardiac effects of low doses of ionizing radiation is highly relevant in the context of interventional cardiology and radiotherapy. Epidemiological data report that low doses of irradiation to the heart can result in significant increase in the cardiovascular mortality by yet unknown mechanisms. In addition co-morbidity factor such as hypertension or/and atherosclerosis can enhance cardiac complications. Therefore, we explored the mechanisms that lead to long-term cardiac remodelling and investigated the interaction of radiation-induced damage to heart and cardiovascular systems with atherosclerosis, using wild-type and ApoE-deficient mice.

**Methods and Results:**

ApoE−/− and wild-type mice were locally irradiated to the heart at 0, 0.2 and 2 Gy (RX). Twenty, 40 and 60 weeks post-irradiation, echocardiography were performed and hearts were collected for cardiomyocyte isolation, histopathological analysis, study of inflammatory infiltration and fibrosis deposition. Common and strain-specific pathogenic pathways were found. Significant alteration of left ventricular function (eccentric hypertrophy) occurred in both strains of mice. Low dose irradiation (0.2 Gy) induced premature death in ApoE−/− mice (47% died at 20 weeks). Acute inflammatory infiltrate was observed in scarring areas with accumulation of M1-macrophages and secretion of IL-6. Increased expression of the fibrogenic factors (TGF-β1 and PAI-1) was measured earlier in cardiomyocytes isolated from ApoE−/− than in wt animals.

**Conclusion:**

The present study shows that cardiac exposure to low dose of ionizing radiation induce significant physiological, histopathological, cellular and molecular alterations in irradiated heart with mild functional impairment. Atherosclerotic predisposition precipitated cardiac damage induced by low doses with an early pro-inflammatory polarization of macrophages.

## Introduction

Epidemiological reports clearly show that cardiac exposure to high doses of ionizing radiation after radiotherapy increase the risk of cardiovascular disease in cancer patients (for instance, in left sided breast cancer patients the heart dose can range from 3 to 17 Gy with an increased risk of cardiovascular death equal to 44%) [1]; [2]; [3]; [4]. Alteration of cardiac function with a decrease in ejection fraction (EF) suggestive of heart failure was also reported in patients who developed long-term radiation-induced cardiac toxicity either after exposure to intermediate dose of ionizing radiation to the heart (<3 Gy) [3] and/or chemo-induced heart toxicity after exposure to anthracyclines [5]. Although the potential risk of late cardiac disease after exposure to low radiation doses was raised a long time ago by the analysis of mortality from cancer and non-malignant diseases among Japanese A-bomb survivors [6]; [7], controversies are still ongoing and biological evidence remains scarce. Mortality from myocardial infarction more than 40 years after radiation exposure was significantly increased in victims who had received an acute total body dose of 1 to 2 Gy. Other data are accruing that both environmental and occupational low-dose exposure may lead to increased risk of cardiac disorders [8]. However, studies conducted in Canadian, British and German nuclear workers showed no evidence of enhanced cardiovascular disease (CVD) [9]; [10]; [11]. The dose threshold and latency time for CVD development after low dose exposure is unknown as well as the pathogenic features and mechanisms of the disease. The enormous latency time (≥15 years) required before occurrence of any measurable symptoms [3];[12] makes the disease difficult to study in humans and co-morbidity factors inevitably influence final outcome.

The establishment of an experimental model dedicated to study heart response to low dose of ionizing radiation constituted the first part of the present study. As cardiovascular co-morbidity such as atherosclerosis is present in >20% of cancer patients [13], we investigated cardiac response in pro-atherogenic ApoE-deficient mice [14]. Finally, several questions were addressed: i) the impact of low doses of ionizing radiation on cardiac function, ii) the time course of the pathogenic development if any, iii) and potential structural and cellular alterations associated. Functional studies along with structural, cellular and molecular characterization allowed us to document for the first time that low doses of irradiation induce cardiac lesions and remodelling that are amplified in a pro-atherogenic genetic background with mild but measurable functional impact. The development of post-irradiation cardiac pathology is largely amplified by aging factors and structural alterations consistent with ongoing scarring and fibrogenic processes. The pathological picture was enhanced and more precocious in ApoE−/− as compared with wild-type (wt). However, in both strains, cardiac fibrosis was associated with inflammatory infiltration that was further characterized.

Today the role of macrophages in cardiac remodelling is well recognized and M1 *versus* M2-polarization is thought to drive the balance between exacerbation of tissue damage (M1) or protection/recovery but possibly fibrogenesis (M2) [15]; [16]; [17]; [18]. Interestingly, a role for macrophages after total body exposure to low dose irradiation has been suggested [19] but macrophage polarization has never been characterized. These long-term changes in the micro-environment and persistent inflammation might alter the tissue and contribute to long-term defects and to chronic release of fibrogenic growth factors [17]. Amongst them a key role for TGF-β1 signalling has been shown, by us and others, in the constitution of radiation-induced fibrotic tissue [20]; [21]; [22]; [23]; [24]. TGF-β1 is also an important mediator of cardiac remodelling and cardiomyocyte hypertrophy [25], its contribution after exposure to low dose of ionizing radiation has been shown in mammary epithelium [26], but has not been previously investigated in the heart. Given the cellular features observed in irradiated hearts at low dose, we hypothesized that both precocious macrophage polarization and earlier TGF- β1 activation could provide the molecular basis for ApoE−/− enhanced sensitivity to low dose of ionizing radiation.

## Materials and Methods

### Animals and irradiation procedures

A total of 90 male C57Bl6 (Charles River Laboratories, Research Models and Services, Germany GmbH) and 63 homozygous male ApoE deficient mice inbred in C57Bl6 background were purchased from Charles River Laboratories (Research Models and Services, Germany GmbH) aged 8±1 weeks were used in all experiments. They were immobilized (without anesthesia) in specially designed jigs and locally irradiated using a YXLON MG325 device (Yxlon International X-ray GmbH, Germany) operated at 200 kV, with a tube current of 20 mA and a beam filter of 0.6 cm Cu, resulting in a dose rate of 0.8045 Gy.min-1. Single local heart doses of 0.2 and 2 Gy, were applied and control groups were sham-irradiated. The exact position of the heart before irradiation was assessed by radiography. Whole heart was irradiated including about 20% of the lung, whereas the rest of the animal was shielded with lead plates. Aged-matched sham (0 Gy) animals were included and compared with irradiated groups at each time point (7–10 mice per dose and time point). Animals were irradiated in Dresden, shipped to Villejuif 1–2 months after irradiation and maintained on a regular chow diet (UAR, France). This study was carried out in strict accordance with the recommendations in the Guide for the Care and Use of Laboratory Animals of the National Institute of Health USA. The protocol was approved by the Committee on the Ethics of Animal Experiments of Landesdirektion Dresden: (file no. 24-1968.1-11/2009-10; Germany); Ministry of Agriculture (Act No. 87-848, 19th of October, 1987; France):and directive 2010/63/EU of the European parliament.

### Echocardiography

Measurement of heart's physiological parameters was done by Trans-thoracic echocardiographic M-Mode, using an ultrasonographic system (Aplio, Toshiba) with a 14 MHz transducer under isofluorane anesthesia (0.75% to 1.0% in oxygen) with spontaneous ventilation. Body temperature of mice was maintained with a heating pad. Left ventricle (LV) diameters at end-diastole (LVEDD) and end systole (LVESD) were measured according to the American Society of Echocardiography leading edge method from TM mode parasternal long axe view. [27];[28];[29].

### Histopathological analysis

One week after hemodynamic studies, mice were killed by cervical dislocation; hearts were collected at dedicated time points. Each heart's weight was measured and normalized with body weight or with tibia length, in order to minimize variations. Organs were fixed in Finefix (Milestone medical, Italy), paraffin embedded and cut into 4 µm sections. Cross sections were taken from the midventricular plane of the heart, and from the plane between the mid-ventricle and base of the heart. Sections were stained with Hematoxilin-Eosin-Saffranin (HES), Sirius Red (SR) and examined using conventional light microscopy and quantified using HISTOLAB software.

### Cardiomyocytes surface area

Mean cardiomyocytes surface areas was determined by measuring 180–200 cardiomyocytes. Images from 4 serial slides of the heart were acquired using a Leica DMR microscope (40× objective) and analysed using HISTOLAB software.

### Protein isolation and Western blotting

Cardiomyocytes (CM) were isolated from ventricular tissue of sham-irradiated and irradiated C57Bl6 and ApoE−/− mice using Cellutron method (Cellutron Life Technology) (∼0.6 million cells per heart). CM were lysed in RIPA buffer containing protease and phosphatase inhibitors (Roche) for western blotting. Sampling was done 20, 40 and 60 weeks after irradiation.

Immunodetection by Western-blot was performed by electrophoresis of proteins in a 12% or 4–12% tris-HCL SDS-PAGE, transferred to PVDF membranes (Biorad). Membranes were blocked with TBS-Tween 0,1%- BSA 5%(sigma) and incubated with primary antibodies included anti-TGF-β1 (1∶500; Millipore); anti-smad 7 (1∶500;Santa Cruz); anti-PAI-1 (1∶500;santa cruz); anti-α-actin sarcomeric (1∶250; SIGMA,). Membranes were incubated with corresponding HRP secondary conjugated antibody (GE Healthcare Life Sciences; diluted at 1∶5000 in TBST containing 2% BSA). Reactive proteins were visualized by chemiluminescence detection system. Images were acquired using ImageQuant imagers coupled to advanced Fujifilm CCD (charge-coupled device) cameras (GE Healthcare Life Sciences). Incubation with rabbit monoclonal α-actin sarcomeric (1∶1000;sigma-aldrich) was performed to normalize the chemiluminescence levels and exposure times.

### ELISA

IL-6 concentration was determined in whole heart lysate using the mouse IL-6 antibody array kit (RayBio Mouse IL-6 ELISA, Raybiotech, Inc., USA). Minimum sensitivity of this assay is <3 pg/ml and measurement is performed 450 nm using a Microplate Manager (Biorad).

### Immunofluorescence

Serial slides (4 µm) were examined for inflammatory cells quantification of macrophages and leukocytes. For macrophage quantification, double immunofluorescence was performed using CD68 rabbit polyclonal antibody (diluted at 1∶50 in TBST 3% BSA, H-255: sc-9139, Santa Cruz Biotechnology, inc.) and purified rat anti-mouse CD11b (diluted at 1∶100 in TBST 3% BSA, 557394, BD Biosciences). Leukocytes were typed *in situ* using purified rat anti-Mouse CD45R (diluted at 1∶20 in TBST 3% BSA, RA3-6B2, BD Pharmingen). Corresponding secondary conjugated antibody (respectively anti-goat Alexa Fluor 488 and 568, Invitrogen) were diluted at 1∶2000 in TBST containing 3% BSA. Stained tissue sections were examined using a confocal microscope equipped with a JVC color video camera coupled to an imaging analysis system (Zeiss). The whole sections were evaluated for number of positively stained cells for CD45R, CD68 and CD11b and the positivity was calculated as the number of positive cells per mm^2^ tissue area.

### Statistical analysis

Statistical analysis for survival curves was performed using Mantel-Cox test. Other data were expressed as Mean ± SEM and analyzed using the ANOVA and the Student Newman Keul's test.

## Results

### Increased sensitivity of ApoE−/− mice to low dose irradiation

**Figure 1:**
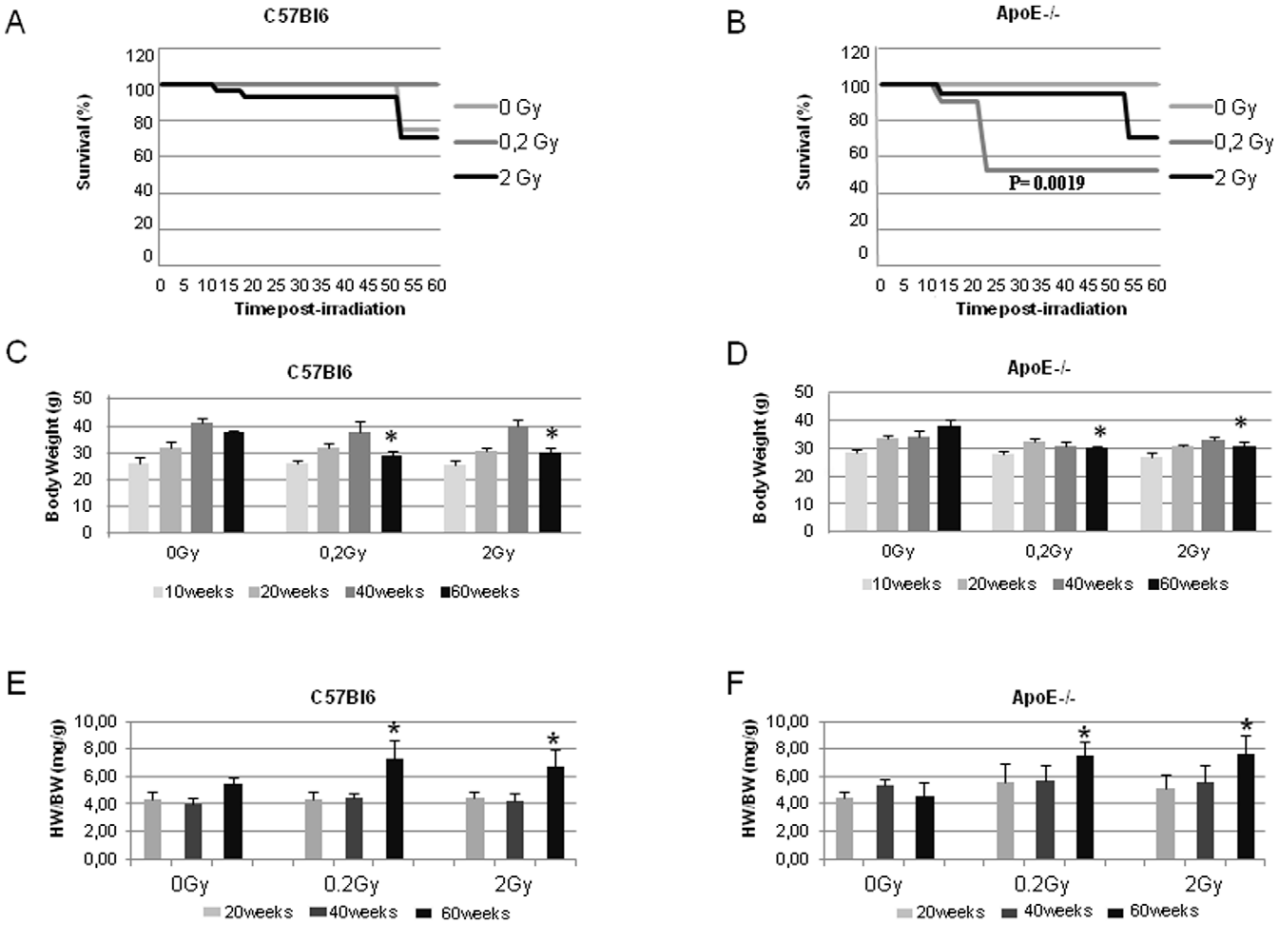
Survival curve, weight and heart weight of mice post-irradiation. **A** and **B** Survival curves of C57Bl6 and ApoE−/−Statistical analysis by Mantel-Cox test: ApoE−/− irradiated mice at 0.2 Gy *versus* age-matched controls P = 0.0019. **C** and **D** Weight of C57Bl6 and ApoE−/−. **E** and **F** weight of heart (2 ventricles)/weight of mice post-irradiation. * P<0.05; 0 Gy vs irradiated groups at 60weeks. Each group comprised of 7 to 10 mice.

Irradiation has no significant impact on long-term survival (60 weeks) of C57Bl6 (the mortality observed is inherent to long-term studies) (Figure 1A). Conversely, ApoE−/− exhibited enhanced radiation sensitivity to low dose radiation (0.2 Gy) associated with 47% of death around 20 weeks post-irradiation (Figure 1B).

In C57Bl6, weight increased linearly with aging uptil 40 weeks post-irradiation and then significantly dropped between 40 and 60 weeks; suggesting late development of pathological process (Figure 1C). The weight of ApoE−/− was initially higher with a continuous weight up-take in non-irradiated animals whereas weight up-take slowed-down in the irradiated groups and was significantly lower at 60 weeks after irradiation (Figure 1C and 1D).

### Cardiac physiological parameters are altered by exposure to low dose irradiation

Cardiac function was assessed by echocardiography and was performed in order to keep the heart rate as close as possible to physiological levels (Table 1).

**Table 1:** The measurements of cardiac physiologic parameters: Heart rate, Ejection fraction (EF), Shortening fraction (FS), left ventricular end-diastolic dimension (LVEDD) as well as the interventricular septal thickness in diastole (IVSd), LV posterior wall thickness (LVPWd) and LVmass/Body weigh (LV mass/BW).

	*20 weeks post-irradiation*			*40 weeks post-irradiation*			*60 weeks post-irradiation*		
*C57Bl6*	*0 Gy*	*0.2 Gy*	*2 Gy*	*0 Gy*	*0.2 Gy*	*2 Gy*	*0 Gy*	*0.2 Gy*	*2 Gy*
**Heart rate (bpm)**	**511.0±60.8**	**480.5±35.0**	**549.8±33.7**	**459.2±28.4**	**451.0±46.9**	**461.7±38.9**	**492.7±27.7**	**463.0±33.5**	**473.0±21.5**
**EF (%)**	**83.9±3.7**	**59.1±8.8^§§^**	**70.4±9.0^§^**	**70.11±7.0**	**54.01±10.5^†^**	**56.95±7.7^†††^**	**72.8±3.07**	**58.8±5.8** ^‡^	**54.1±6.7^‡^**
**FS (%)**	**49±8.0**	**31.4±6.2^§§^**	**39.3±7.2^§§^**	39.2±5.8^*^	**27.8±6.5^†^**	**29.6±5.5^†††^**	**41.2±2.6**	**33.9±7.6**	**27.7±4.2** ^‡‡^
**LVEDD (mm)**	**3.1±0.4**	**4.4±0.3^§§§^**	**3.6±0.3^§^**	3.7±0.3^**^	**4.1±0.4^†^**	**4.1±0.3^†^**	3.8±0.2^**^	**3.9±0.5**	**4.1±0.2** ^‡^
**IVSd (mm)**	**0.9±0.2**	**0.8±0.2**	**1.0±0.1**	**0.9±0.1**	**1.1±0.2^†^**	**1.1±0.2^†^**	1.2±0.3^*^	**1.1±0.2**	**1.2±0.5**
**LVPWd(mm)**	**1.0±0.2**	**0.9±0.2**	**1.1±0.2**	1.3±0.2^**^	**1.1±0.2**	**1.3±0.5**	1.3±0.2^**^	**1.1±0.2**	**1.3±0.1**
**LV mass/BW (mg/g)**	**4.00±0.8**	**8.1±1.2^§^**	**6.3±0.7^§^**	**5.0±0.6**	**7.0±0.7^†^**	**7.1±0. 7^†^**	6.7±0.6^**^	**8.2±0.3** ^‡^	**9.8±1.1** ^‡^

*ApoE−/−*	*0 Gy*	*0.2 Gy*	*2 Gy*	*0 Gy*	*0.2 Gy*	*2 Gy*	*0 Gy*	*0.2 Gy*	*2 Gy*
**Heart rate (bpm)**	**498.6±64.7**	**538.6±49.6**	**479.2±45.7**	**500.2±22.4**	**472.8±34.7**	**465.0±40.0**	**474.5±11.7**	**519.5±50.5**	**490.7±41.0**
**EF (%)**	**73.3±5.8**	**59.9±3.7^§§§^**	**53.7±9.6^§§^**	**74.4±6.9**	**62.1±8.3^††^**	**63.0±5.9^†^**	**75.3±5.7**	**57.5±8.9** ^‡^	**58.7±8.3** ^‡‡^
**FS (%)**	**41.6±5.6**	**31.4±2.5^§§^**	**27.5±6.8^§§^**	**42.4±6.3**	**32.9±5.7^†^**	**33.8±4.3^†^**	**43.5±5.0**	**30.0±5.9** ^‡^	**30.7±5.4** ^‡‡^
**LVEDD (mm)**	**3.5±0.5**	**3.9±0.3**	**4.0±0.3^§^**	**3.2±0.1**	**3.6±0.3^††^**	**4.0±0.4^†††^**	**3.6±0.3**	**4.1±0.6**	**3.8±0.2**
**IVSd (mm)**	**1.0±0.1**	**1.0±0.1**	**1.0±0.1**	1.2±0.2^*^	**1.2±0.1**	**1.1±0.1**	1.2±0.1^**^	**1.0±0.1** ^‡‡^	**1.0±0.1** ^‡‡^
**LVPWd(mm)**	**1.2±0.4**	**1.0±0.2**	**1.0±0.2**	**1.2±0.2**	**1.2±0.2**	**1.0±0.2**	**1.2±0.2**	**0.9±0.1** ^‡‡^	**1.0±0.2**
**LV mass/BW (mg/g)**	**3.9±0.9**	**4.9±0.6**	**5.0±0.9**	**4.1±0.3**	**5.4±0.6^†^**	**6.1±0.5^††^**	**3.4±0.6**	**5.5±0.9** ^‡^	**4.3±0.7**

Sham 20weeks compared by sham groups at each time point ^*^p≤0.05; ^* *^p≤0.01;^***^p≤0.001.

0 Gy at 20weeks vs irradiated groups at this time point **^§^** p≤0.05; **^§§^**p≤0.01; **^§ § §^**p≤0.001.

0 Gy at 40weeks vs irradiated groups at this time point ^†^ p≤0.05; ^††^p≤0.01; ^†††^p≤0.001.

0 Gy at 60weeks vs irradiated groups at this time point ^‡^ p≤0.05; ^‡‡^p≤0.01.

n = 7 animals in each group (20 and 40 weeks post-irradiation and n = 5 animals in each group (60 weeks post-irradiation).

The latest time-points of our study (40 and 60 weeks) revealed aging in non-irradiated C57Bl6 with LV hypertrophy without significant LV dysfunction that was not observed in ApoE-deficient animals. In C57Bl6, 0.2 and 2 Gy induced a dilated cardiomyopathy with eccentric hypertrophy and LV dysfunction as early as 20 weeks post-irradiation. The progression of LV dysfunction remained stable over the 60 weeks duration of the study (Table 1). In ApoE−/−, LV dysfunction was observed 20 weeks post-exposure to 0.2 Gy and mild hypertrophy appeared at 40 and sustained at 60 weeks, however no significant LV dilatation was observed. Two Gy induced a marked LV dysfunction in ApoE−/−, significantly greater than in wt-C57Bl6, particularly 20 weeks post-irradiation. In addition, both strains exhibited altered LV contractility with a mild although significant decrease in ejection and shortening fraction after exposure to both 0.2 and 2 Gy (Table 1). Both strains developed bilateral ventricular hypertrophy as evidenced by gravimetry at 60 weeks (Figure 1E and 1F).

### Cardiomyocyte hypertrophy induced by low dose irradiation occurs earlier in Apo E−/−

**Figure 2:**
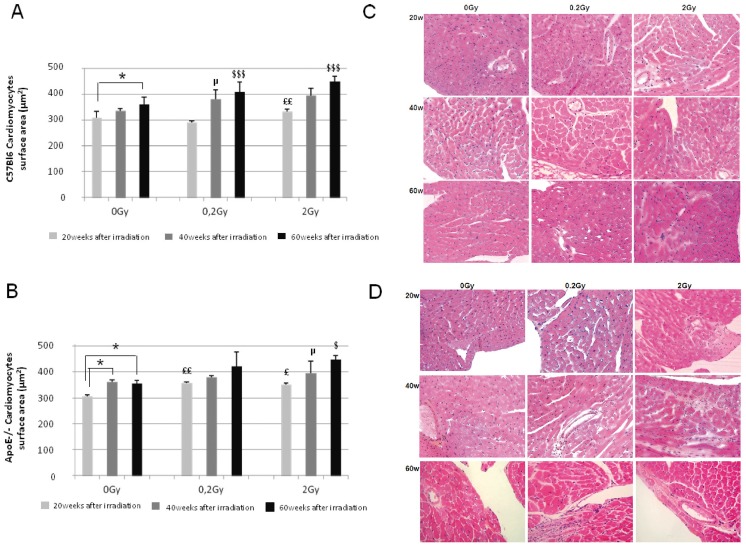
Histological analysis of control and irradiated hearts 20, 40 and 60weeks post-irradiation. **A** and **B.** Cardiomyocyte surface area measurement was performed on 180–200 cardiomyocytes from 4 serial slides (HES heart sections) of C57Bl6 and ApoE−/− respectively (n = 3–5 animals in each group), 0 Gy 20weeks vs 0 Gy 40weeks; * P<0.05; 0 Gy 20weeks vs 0 Gy 60weeks; * P<0.05; 0 Gy vs irradiated groups at 20weeks; £ P<0.05, ^££^ P<0.01; 0 Gy vs irradiated groups at 40weeks; ^μ^ P<0.05, ^μμ^ P<0.01, 0 Gy vs irradiated groups at 60weeks; ^$^ P<0.05, ^$$$^ P<0.001. **C** and **D.** Histological assessment of cardiac ventricular pathology by HES staining in C57Bl6 and ApoE−/− respectively (n = 4–6 animals in each group and 2 sections of heart per animal). Original magnification ×400, **C** in C57Bl6 and **D** in ApoE−/−.

In C57Bl6, both aging and ionizing radiation induced cardiomyocyte hypertrophy (Figure 2A) and correlated with a significant increase in LV mass (Table 1). After exposure to 0.2 Gy, hypertrophy was significant at 40 weeks and further enhanced 60 weeks post-irradiation. Exposure to 2 Gy accelerated the process that was measurable 20 weeks post-irradiation and worsened 40 and 60 weeks after irradiation (Figure 2A). In ApoE−/−, cellular hypertrophy occurred earlier (20 weeks post-irradiation) suggesting a greater sensitivity of ApoE−/− animals to low dose irradiation. After exposure to 0.2 Gy, increase in cardiomyocyte surface area was significant 20 weeks after irradiation and sustained 40 and 60 weeks post-irradiation (Figure 2B). The effect of higher irradiation dose was similar to that of low dose. Aging was a confounding factor at delayed time points in both strains of mice.

### Ionizing radiation induces dose and time-dependent cardiac structure alteration

In C57Bl6 mice exposed to 0.2 Gy and 2 Gy, the main histopathological alteration was infiltration of inflammatory cells (Figure 2C) that occurred 20 weeks post-irradiation and worsened at 40 weeks. Inflammatory infiltration was observed in inter-cardiomyocyte connective tissue, epicardium and perivascular interstitium. Cardiomyocytes exhibited enlarged nuclei and large spaces between cardiomyocytes were observed, suggesting radiation-induced alteration of cell-to-cell contact. At 60 weeks post-irradiation at 2 Gy, vacuoles were seen in endocardial myocytes (Figure 2C).

In irradiated ApoE−/− (Figure 2D) more severe inflammatory infiltration was observed with an earlier occurence. In addition, interstitial oedema was associated with damaged cardiomyocytes (showing loss of nuclei and vacuoles). At 60 weeks post-irradiation, leukocytic infiltrates a majority of neutrophils was observed. Large areas of replacement fibrosis were more obvious with 2 Gy than at 0.2 Gy, associated with patchy inflammatory zones and vacuolized cardiomyocytes.

### ApoE deficiency promots M1-polarization after low dose radiation exposure

**Figure 3:**
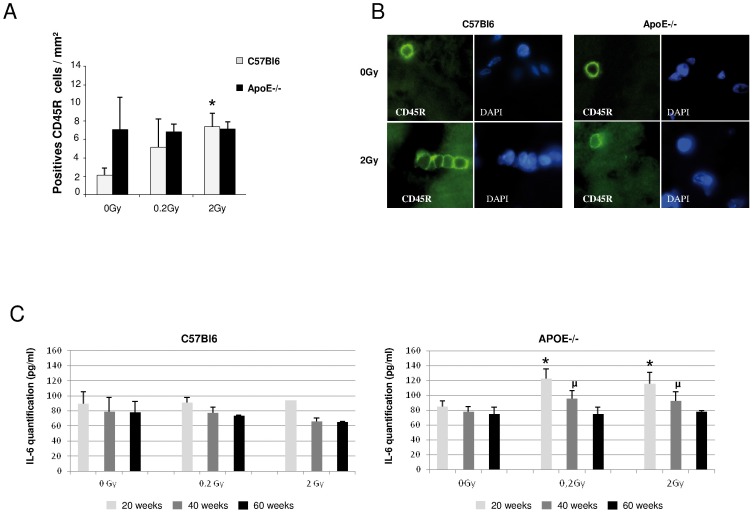
Leucocytes characterization and IL-6 Expression. **A:** Quantitative analysis of the number of leucocytes per mm^2^ tissue section area in C57Bl6 and ApoE−/− mice 40weeks post-irradiation; C57Bl6 0 Gy vs irradiated groups,*P<0.05 (n = 2 sections per heart and 3–5 animal per group and each time point). **B**: CD45R immunofluorescence (green) and DAPI staining (blue) at 0 and 2 Gy, 40weeks post-irradiation (original magnification ×1000). **C.** IL-6 ELISA in cardiac tissues from ApoE−/− and C57Bl6 mice, 20, 40 and 60weeks post-irradiation at 0, 0.2 and 2 Gy. ApoE−/− 0 Gy vs irradiated groups at 20weeks,*P<0.05; ApoE−/− 0 Gy vs irradiated groups at 40weeks; ^μ^P<0.05. (n = 4–6 samples).

**Figure 4:**
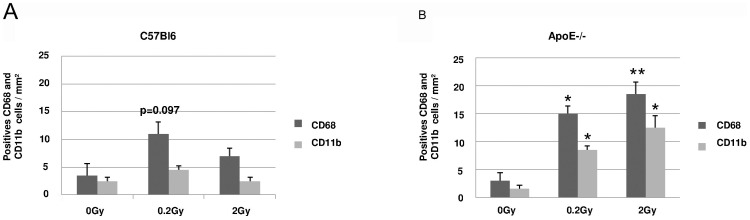
Macrophages typing and quantification. **A** and **B**: Quantitative analysis of number of positive CD68 and CD11b macrophages per mm^2^ heart tissue area in C57Bl6 and ApoE−/− mice respectively (n = 2 sections of heart per animal and 3–5 animals per each group and each time point); ApoE−/− 0 Gy vs irradiated groups at 40weeks,*P<0.05;**P<0.01.

As infiltration of macrophages and inflammatory cells was obvious in histological sections, we thought to phenotypically characterize these infiltrating cells. A significant increase in CD45R+ leukocyte infiltration was observed in C57Bl6, 40 weeks post-irradiation, whereas in ApoE−/− (0 Gy) the baseline level was high and remained stable after exposure to low and medium doses of radiation (Figure 3A and 3B). The most interesting result came from macrophage phenotyping (Figure 4). A general macrophage marker, CD68, was used to identify macrophages *in situ*. An increased number of CD68-positive macrophages was found in ApoE−/− and in C57Bl6 but to a lower extent. In addition, the number of CD11b-positive macrophages, thought to tag M1 polarization, was increased in ApoE−/− irradiated with 0.2 Gy and 2 Gy at 40 weeks post-irradiation, but not in C57Bl6 mice (Figure 4A and 4B). Globally, in C57Bl6, the number of CD11b-positive macrophages was low and not induced by irradiation (Figure 4A). These results show a difference in macrophage polarization induced by irradiation in ApoE−/− *vs* C57Bl6 animals and suggest that the pro-inflammatory status of ApoE deficient mice is prone to cardiac damage. This hypothesis was further supported by IL6 induction found in ApoE deficient mice 20 and 40 weeks post-irradiation with 0.2 and 2 Gy (Figure 3C).

### Cardiac fibrosis is induced by low dose irradiation and enhanced in ApoE−/− mice

**Figure 5:**
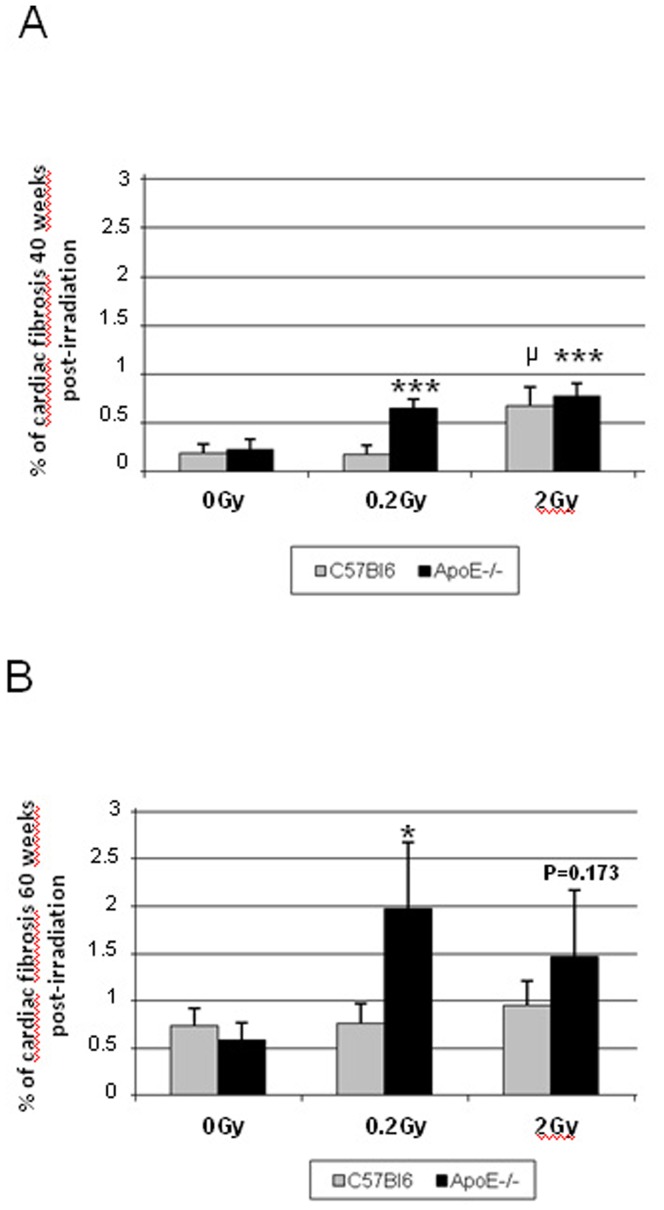
Quantification of cardiac Fibrosis. Two sections of heart stained with Sirius Red and examined to quantify the percentage of cardiac fibrosis. **A:** Fibrosis quantification in ApoE−/− and C57Bl6 mice 40 weeks post-irradiation **B:** Fibrosis quantification in ApoE−/− and C57Bl6 mice 60weeks after irradiation. Non-irradiated ApoE−/− vs irradiated ApoE−/− *p<0.05;*** p<0.001 and non-irradiated C57Bl6 vs irradiated C57Bl6 ^μ^p<0.05. (n = 4–6 animals in each group and 2 sections of heart per animal).

In C57Bl6, 40 weeks post-irradiation with 0.2 Gy, collagen deposition was essentially peri-vascular, and was additionally found in the epicardium and interstitium at 2 Gy (data not shown). At 60 weeks post-irradiation, perivascular fibrosis worsened at 0.2 Gy and more collagen was observed in the right ventricle. In the 2 Gy group, a thin and continuous fibrotic layer was observed all along the pericardium. Fibrosis quantification showed an increase in collagen deposition after a single dose of 2 Gy at 40 and 60 weeks post-irradiation (Figure 5A and 5B).

Non-irradiated ApoE−/− had a richer interstitium, with more collagen deposition around vessels and thicker epicardium than C57Bl6 (data not shown). After irradiation, collagen deposition was perivascular and interstitial at 20 weeks post-irradiation with 0.2 Gy. At forty weeks post-irradiation, there was endocardial, epicardial and peri-vascular fibrotic infiltration. At 60 weeks post-irradiation, the fibrotic picture was massive. Scars were more apparent after 2 Gy irradiation rather than 0.2 Gy, showing large areas of replacement fibrosis, patchy inflammatory zones and vacuolized cardiomyocytes (data not shown).

Fibrotic deposition was significantly enhanced in ApoE−/− as compared to C57Bl6 (Figure 5A and 5B). Respectively 2.8 and 3.4 fold increase in collagen levels were measured 40 weeks post-irradiation and 3.3 and 2.5 fold increase at 60 weeks in groups irradiated at 0.2 and 2 Gy, *versus* the sham-irradiated group (Figure 5A and 5B). In contrast, in C57Bl6, the increase in collagen level was found to be significant only in the group irradiated at 2 Gy, 40 weeks post-irradiation. No sign of radiation-induced atherosclerosis was observed in hearts of any of the groups. Next, we thought to investigate some of the molecular aspects associated with the enhanced sensitivity of ApoE−/− to low doses of irradiation. These cellular and whole animal data were expected to be underpinned by molecular events, which we next characterized.

### Cardiomyocytes isolated from ApoE−/− exhibit precocious TGF-β1 pathway induction

**Figure 6:**
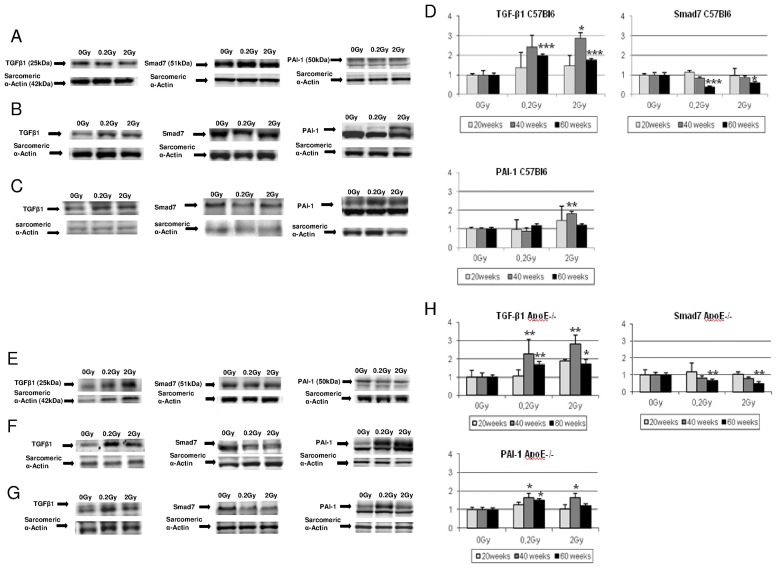
Modulation of TGF-β1 pathway in cardiomyocytes in C57Bl6 and ApoE−/− mice. Western blot were performed with antibodies against TGF-β1 (25 kDa), Smad7 (51 kDa), PAI-1 (50 kDa), and α-actin sarcomeric (42 kDa). In C57Bl6 mice: **A**. 20 weeks post irradiation; **B**. 40weeks post irradiation and **C.** 60weeks post irradiation. **D**: Quantitative analysis of TGF-β1 pathway in cardiomyocyte lysate isolated from non-irradiated and irradiated C57Bl6mice 20, 40 and 60weeks post-irradiation. In ApoE−/− mice: **E**.20 weeks post irradiation; **F**. 40weeks post irradiation and **G**. 60weeks post irradiation. **H.** Quantitative analysis of TGF-β1 pathway in cardiomyocyte lysate from non-irradiated and irradiated ApoE−/− mice 20, 40 and 60weeks post-irradiation. At each time point non-irradiated are compared with irradiated groups *P<0.05 **P<0.01, ***P<0.001.(N = 2–4 animals per conditions and 4–6 samples of cardiomyocytes per animal).

TGF-β1 signalling in response to low doses of irradiation was investigated by western blotting in cardiomyocytes isolated from irradiated or non irradiated C57Bl6 and ApoE−/− mice, 20, 40 and 60 weeks post-irradiation (Figure 6). Heart exposure to 0.2 Gy led to enhancement of fibrogenic factors in cardiomyocytes isolated from ApoE−/− 40 weeks post-irradiation (Figure 6F and 6H) whereas similar induction occurred but was delayed until 60 weeks in C57Bl6 (Figure 6C and 6D). In ApoE−/− mice irradiated at 0.2 Gy, significant expression of TGF-β1 with was observed concomitantly with enhanced expression of PAI-1 40 and 60 weeks post-irradiation (Figure 6F, 6G and 6H); exposure to 2 Gy lead to the induction of TGF-β1 and PAI-1 in both strains, 40 weeks post-irradiation (Figure 6B, 6D, 6F, and 6H). These results correlated with fibrosis score (figure 5A). Smad7 expression was repressed in both strains of mice 60 weeks post-irradiation suggesting an additional inhibition of TGF-β1 inhibitory pathway during the late phase of the pathology (Figure 6C, 6D, 6G and 6H).

## Discussion

The present experiments were designed to investigate the possible development of delayed cardiac disease after exposure to low and intermediate doses of ionizing radiation in wt-C57Bl6 and ApoE-deficient mice, to assess the contribution of the atherogenic co-morbidity factor. The main conclusions are: i) Mild but significant alteration in cardiac function occurred after exposure to low doses of ionizing radiation consistent with development of eccentric hypertrophy; ii) Structural lesions are reported with cardiomyocyte hypertrophy, macrophage infiltration and cardiac fibrosis, associated with release of TGF β1 and effectors such as PAI-1; and iii) The atherogenic susceptibility in ApoE deficiency was shown to be a risk factor for radiation-induced cardiac disease after low dose ionizing radiation, although no atherosclerotic remodeling was observed in irradiated hearts but was reported in large vessels [14]. In addition, a mechanism is proposed in which ApoE-deficiency would promote pro-inflammatory profile in cardiac tissue.

In the present study, long-term maintenance of overall cardiac physiological function was observed after low dose irradiation, with alterations scored as Class I by the International Small Animal Cardiac Health Council (ISACHC). However, mild decreased ejection fraction (EF) and shortening fraction (FS) were seen, consistent with clinical data [5] and with other *in vivo* experimental data [30], [31]. These observations are not consistent with other experimental reports obtained in rats and mice after exposure to high dose of ionizing radiation and in which increase in EF and SF was shown [32];[33]. The significance of this discrepancy between the effect induced by high and low doses of irradiation remains to be investigated but in any case, the reported variation remains in the physiological range, suggesting occurrence of compensatory mechanisms. We characterized part of these compensatory mechanisms, with increased expression of TGF-β1 and PAI-1 linking with the observed radiation-induced hypertrophy, fibrogenesis and contractile dysfunction of cardiomyocytes [34], [30], [35]. Contribution of TGF-β1 to compensatory mechanisms has already been reported after myocardial infarction [36]; [25] as well as PAI-1 secretion by cardiomyocytes and mast cells [37]; [36]. The relevance of TGF-β1 and PAI-1 is further supported by a recent clinical study in which variants of TGF-β1 and PAI-1 genes were shown to be possible risk factors for cardiovascular disease in patients after radiotherapy for breast cancer [38].

Strain-specific differences were reported between C57Bl6 and ApoE-deficient mice with ApoE−/− exhibiting enhanced sensitivity to low doses of irradiation as compared to C57Bl6. ApoE−/− show enhanced subacute mortality, increase structural alterations, more inflammation and enhanced fibrotic deposition consistently with Gabriels *et al*. results [39]. Interestingly this hypersensitivity tends to be lost at intermediate doses and is totally erased at high doses where severe cardiac damages and delayed mortality occurred (data not shown). As the ApoE−/− strain used was inbred with a C57Bl6 background, we attributed the enhanced low dose sensitivity to ApoE deficiency. Our observations are consistent with a recent report by Mitchel et *al*. that described a significant impact of low doses of irradiation on the development of atherosclerosis in ApoE−/− mice [19]. For instance, radiation-induced carcinogenesis induced by exposure to low doses is known to be highly dependent upon genetic profile (for instance p53, BRCA, PARP status) [40]; [41] and today, the proportionality between mean organ dose and the risk of radiation-induced cancer is well established. In contrast to radiation-induced secondary cancer, cardiovascular complications are of multicellular origin and epidemiological data show that a linear dose risk relationship cannot apply. Our observations showing no direct dose-response in cardiac effects seems in accordance with epidemiological studies conducted in the Japanese A-bomb survivors. In addition, a window of hyper-radiosensitivity (defined as HRS) to low radiation doses has been described *in vitro* [41]. This HRS process cannot be predicted by back-extrapolating the cell survival response from higher doses and had never been properly investigated *in vivo*. Two hypotheses have been proposed to explain HRS *in vitro*. The greater amount of injury produced by larger doses is first above a putative damage-sensing threshold for triggering faster DNA repair; secondly it causes changes in DNA structure or organization that facilitates constitutive repair. *In vivo* the scenario is probably even more complex but several explanations can be proposed:

1. **Low dose of irradiation promotes M1-polarisation in ApoE−/− mice.** Apolipoprotein E (ApoE) is anti-inflammatory and its deficiency causes all features of atherosclerosis mediated by acute inflammation. In our model no atherosclerotic lesions were observed in the heart after exposure to 0.2 Gy but were seen in large vessels; in addition, macrophage infiltration was seen. Macrophages were already identified as pathogenic mediators of atherosclerotic responses to low doses of ionizing radiation in ApoE−/− [19]. ApoE is known to promote macrophage conversion from the pro-inflammatory M1 to anti-inflammatory M2 phenotype [42] and the enhanced M1-polarization associated with high IL-6 level reported here is consistent with this. As M1-polarization was reported to trigger cardiac hypertrophy [18], we propose that cardiomyocyte hypertrophy would be driven by M1-polarization in irradiated ApoE−/− with subsequent activation of fibrogenic pathways and fibrotic deposition would then occur.
2. **Low dose irradiation altered cardiomyocyte structure and function.** Twenty weeks post-exposure with 0.2 Gy, premature death of ApoE−/− mice was observed and associated with alteration of cardiomyocyte morphology in the survivors. At this time point, cardiomyocytes exhibited enlarged nuclei, cytoplasmic vacuoles and a loss of cell-to-cell contact. All these structural alterations may cause muscle fiber dystrophy and left ventricular dysfunction that may lead to death of the animals, as described in mouse models of ischemia and myocardial infarction [43]; [44].
3. **Low dose irradiation altered atrial function.** A recent report showed TGF-β1 and IL-6 over-expression by locally recruited macrophages in fibrillating atria [45]. Both cytokines are known to affect contractility and electrical stability of myocytes and may enhance the risk of arrhythmia [46]; [34]. To establish this with certainly, telemetric analysis is required, but arrhythmia may explain the sudden death of ApoE−/− deficient animals 20 weeks post-irradiation.

In summary, our results show that cardiac exposure to low dose of ionizing radiation induced significant structural, cellular and molecular alterations in irradiated-heart with a mild but significant functional impairment. According to Seemann et *al.,* irradiation induces stress signals directly by initiating an inflammatory response, associated with progressive structural damage to myocardium and the microvasculature [33]. In addition, atherosclerosis is a co-morbidity factor that enhances and accelerates structural deterioration of the heart in response to low doses of ionizing radiation. Two distinct fibrogenic mechanisms seemed to occur in ApoE−/− and C57Bl6: i) In ApoE−/−, reactive fibrosis seemed to occur in response to inflammatory processes by an increase in inflammatory cytokines such as IL-6 produced by M1-macrophages, followed by a precocious and concomitant increase in specific pro-fibrotic factors (TGF-β1 and PAI-1 40 weeks post-irradiation) and ii) In C57Bl6, reparative fibrosis would occur in interstitial regions in response to loss of cardiomyocytes [47]. The pathogenic outcome is essentially the same but the initiating mechanisms are fundamentally different. However, even if exposure to low dose irradiation affects cardiac structure, cellular and molecular patterns, the heart copes with it for a long time but a slow decline in LV function does occur until a breaking point. Characterization of this breaking point will be the next step, to see whether these modifications will translate into overt clinical disease. Our findings may have an impact on radiation protection rules for patients and population.
